# Study motives, career choices and interest in paediatric dentistry among final year dental students in Nigeria

**DOI:** 10.1186/1472-6920-14-130

**Published:** 2014-07-02

**Authors:** Morenike O Folayan, Oyinkan O Sofola, Mohammad R Khami, Ayodeji O Esan, Bamidele O Popoola, Omolola O Orenuga, Nkiru Folaranmi, Taofeek O Ligali, Abimbola S Phillips

**Affiliations:** 1 Department of Child Dental Health, Obafemi Awolowo University, Ile-Ife, Nigeria; 2 Department of Preventive Dentistry, University of Lagos, Lagos, Nigeria; 3 Research Center for Caries Prevention, Community Oral Health Department, School of Dentistry, Tehran University of Medical Sciences, Tehran, Iran; 4 Department of Preventive and Community Dentistry, Obafemi Awolowo University Teaching Hospitals Complex, Ile-Ife, Nigeria; 5 Department of Child Oral Health, University of Ibadan, Ibadan, Nigeria; 6 Department of Child Dental Health, University of Lagos, Lagos, Nigeria; 7 Department of Child Dental Health, University of Nigeria, Enugu Campus, Nigeria; 8 Department of Preventive and Community Dentistry, University of Maiduguri, Maiduguri, Nigeria; 9 Department of Community Medicine, Obafemi Awolowo University Teaching Hospitals Complex, Ile-Ife, Nigeria

**Keywords:** Study, Motives, Career, Nigeria, Culture, Sex, Paediatric dentistry, Dental students

## Abstract

**Background:**

Students’ motives for studying Dentistry have been a subject of interest for years because of the potential for understanding the psychological makeup and subsequent job satisfaction for the dentist. It is also useful in identifying expectations of the profession. This study therefore tried to identify study motives and career preferences of dental students especially with respect to the practice of paediatric dentistry.

**Methods:**

This was a cross-sectional study using a self-administered questionnaire. The final year students in six dental schools in Nigeria were required to fill the questionnaire. Students were asked to rank their motives and career preferences on a Likert like scale with points ranging from 0–5 where 0 represented a factor that had no influence on their decision and 5 represented a very influential factor. The underlying dimensions for study motives, career preference, impression about and motive for interest in the practice of paediatric dentistry were identified using factor analysis.

**Results:**

One hundred and seventy nine of 223 students (80.3%) participated in this study. Motives for the practice of dentistry included characteristics of the profession, altruism and intellectual challenges, existence of artistic theme in dentistry and parent’s recommendation. Overall, 67.1% of respondents indicated interest in postgraduate studies and 50.8% were interested in paediatric dentistry practice. The main motives for showing interest in the practice of paediatric dentistry were ‘personal interest, professional interest and interest of significant others in children’, and ‘family influence’. Significantly more males than females were interested in the practice of paediatric dentistry though the motives for interest in the practice of paediatric dentistry did not differ significantly by sex or age.

**Conclusion:**

The non-significant sex difference in the motives for interest in the practice of paediatric dentistry is a possible reflection of changes in strong cultural themes in the motives for career choices in Nigeria.

## Background

Students’ motives for studying Dentistry have been a subject of interest for years because of the potential for understanding the psychological makeup and subsequent job satisfaction for the dentist. It is also useful in identifying expectations of the profession. Previous studies in various parts of the world have shown that a majority of the students had “characteristics of the profession” and “social status and security” dimension as their top-ranked motives for studying dentistry [1-4]. Very few students had altruism and intellectual challenges as their first motive dimension. This shows that a desire to serve the public is not among the most important motivations for choosing to study dentistry for many dental students [3]. In a study in Nigeria, interest, prestige, good job opportunities abroad as well as regular work hours were the top ranked motives for studying dentistry [5]. The need for the dental education system to train students who have a sense of social responsibility in view of these findings had been emphasized earlier [3,6,7]. Sex differences have also been reported in students’ study motives and career preferences [5,7-12].

Previous studies have also shown a tendency for the majority of the students to prefer to continue their education towards a specialty degree [7,13-15]. This has implications for the dental workforce planning in any country. While a lot of studies have been conducted in Europe and North America on specialty preference, information is sparse in Nigeria. One study conducted in Nigeria about a quarter of them intended to specialize in oral surgery while 8.6% of them indicated a preference for paediatric dentistry [16].

There is relatively short manpower in paediatric dentistry in Nigeria. At the time of the conduct of this study (2012), Nigeria had 25 practicing paediatric dental specialists with 15 employed in dental training institutions. In effect, there are only 25 practicing paediatric dentists serving a national population of 58,736,297, children under the age of 15 years [17]. This is incomparable to the 4.03 paediatric dental practitioners for every 100,000 US children younger than 18 years of age reported in the US as at 2002 [18]. In 2002, paediatric dentistry had the 5^th^ highest number of trained oral health specialists amongst the nine specialities of training in Dental Surgery by the National Postgraduate Medical College. The top four specialities were Oral and Maxillofacial Surgery (33%), Oral Pathology (16%), Conservative Dentistry (12%) and Orthodontics (9%). Paediatric Dentistry constituted 7% of the specialists trained by the College [19].

Providing oral health care, including specialised oral health care, for this teeming young population is important because of the high oral disease burden in children in Nigeria: the prevalence of caries is high [20] and untreated caries in the primary dentition range from 92% in Ile-Ife to 95.6% in Lagos [20,21].

Manpower development for both basic and specialised dental care is crucial in view of the anticipated increase in awareness and uptake of dental care services following the successful integration of oral health into the primary health care system in line with current plans contained in the new national oral health policy [22]. It is therefore important to identify what motivates dental students to specialise in paediatric dentistry in an effort to drive child care speciality practices in Nigeria, a country with with diverse cultural and socio-economic context when compared to Europe and North America [23].

The present study was conducted in Nigeria. The country has eight dental schools of which six have students in their final year of study. Dental students spend 6 years in dental school: the first 3 years are spent studying basic sciences (anatomy, physiology, pharmacology and biochemistry), one year is spent studying medicine, surgery and pathology, and two years are spent in dental school rotating through ten specialties (paedodontics, orthodontics, oral and maxillofacial surgery, oral pathology, periodontology, oral medicine, community dentistry, restorative dentistry, prosthodontics, and oral diagnosis and oral radiology). In four of the six dental schools, the trainers of students in paediatric dentistry are specialists. The present study plans to identified study motives and career preferences of dental students in their final year of study in dental schools in Nigeria. It also tried to identify factors that motivate students to consider paediatric dentistry as a possible career path. The study is part of a bigger study of which part of the data had been previously published [20].

## Methods

### The target population

The target population were senior dental students (those at the last year of study toward the first degree) of six of the eight dental schools in Nigeria.

### Pilot study

A pilot study was performed in order to revise the contents of the primarily structured questionnaire. Six final year students who were preparing to retake examination papers they had failed during their final examination filled the forms and made suggestions for possible revision of the questionnaire based on ease of understanding of terminologies and its potential conflicting interpretations. The questionnaire was finalized through further discussions with the group.

### Questionnaire administration

A total of 223 questionnaires were administered of which 179 (80.3%) were returned. The questionnaire was distributed to students in their ordinary classroom settings and returned immediately. Response was anonymous. The objectives of the study and the voluntary nature of study participation were described to the students. In addition to sex and age, the questionnaire requested information on the following items:

#### Motives to study dentistry

To assess their motives to study dentistry, the students were asked to give a score from 0 to 5 to each of the 12 given alternatives according to the degree the alternative had influenced their decision to study dentistry. The alternatives were: *Failure to be admitted to other programmes, Parents’ recommendation, Friends’ or relatives’ recommendation, Personal interest in dentistry, Interest in working with one’s hands, Existence of artistic theme in dentistry, High income from practicing dentistry, Ability to be self-employed, Social status of being a dentist, Helping people to improve their health, Playing a role in community health promotion,* and *Being a researcher in dentistry.* The mean of the scores for each alternative was calculated for each alternative in order to rank the motivations. Scores 0, 1 and 2 were categorised as low motives while scores 3, 4 and 5 were categorised as strong motives for each of the motive options. The dichotomy was done to facilitate comparisons between the subgroups.

#### Career preferences

In order to assess the respondents’ career preferences, the students were asked to indicate their willingness to work in each of nine available fields in dentistry after graduation by giving a score from 0 to 5. Interest in each of the career preference was dichotomised as described previously for “Motives to study dentistry”.

#### Impression about the practice of paediatric dentistry

In order to assess their impression about paediatric dental practice, the students were asked to indicate their opinion of peadiatric dentistry through a semantic differential scale comprising 10 qualities and their opposites on a scale from 1 to 7. A score of 5 to 7 was classified as having a positive opinion of paediatric dentistry. Qualities assessed included *usefulness to the community, prestigious, essential, efficient, scientific, attractive, valuable, simple, not costly, and good use of the time of the dentist.*

#### Motives for interest in paediatric dentistry

Only those students who were interested in practising or specialising in paediatric dental practice were asked to rate factors that influenced their decision. Respondents were asked to rate 10 factors giving a score from 0 to 5 to each of the factors, where 0 represented a factor that had no influence on their decision and 5 represented a very influential factor (two or more alternatives can have same scores). Room was given for respondents to list other influential factors and rank them. Scores 0, 1 and 2 was categorised as low while scores 3, 4 and 5 were categorised as strong for each of the motive options.

### Statistical analysis

Chi-square test was used to evaluate the statistical significance of differences in frequencies between subgroups. Sex was categorized as male and female. Age was categorised into three groups based on the terciles. The underlying dimensions for study motives, career preference, impression about the practice of paediatric dentistry and motive for interest in paediatric dentistry were identified using a factor analysis with principle component method and varimax rotation. Factor analysis was used to provide additional insight into basic dimensions around which students’ motives were organized. This helped to give better understanding of the central themes around which students have organized their thinking [10]. Each item that loaded at 0.60 or greater on only one factor was included as an item for a given factor [8,13]. Based on the factor analysis, new variables vis-à-vis each factor was formed by summing the values of the original variables with the highest loadings in that factor. The sum of the variables were then standardized by dividing the sum by the number of variables included [10]. Cronbach’s alpha, a measure of the reliability of a scale was also calculated for the items that made up each. The lowest possible scale value was 1.0 (Low importance) while the highest possible scale value was 5.0 (High importance). Scale means were calculated for males and females, and for the three age categories. Differences in the sex and age scale scores were examined.

### Ethical consideration

Ethics clearance for the study was obtained from the Health Research Ethics Committee of the Obafemi Awolowo University Teaching Hospitals Complex, Ile-Ife, Nigeria. Informed consent was obtained from all participating students. Data was collected without identifiers. All due ethical considerations were given during the conduct of the study, handling of study participants and the handling of the study data.

## Results

### Profile of respondents

The profile of the study respondents had been previously reported [20]. One hundred and seventy-nine students of the 223 eligible students filled the questionnaire giving a response rate of 80.3%. There were 106 (59.2%) men and 71 (39.7%) women. Two (1.1%) respondents did not indicate their sex.

### Motives to study dentistry

Table 1 shows the distribution of respondents’ identified motives for studying dentistry. The top three motives were parent’s recommendation (3.17), ability to be self-employed (3.12) and friend’s/relative recommendation (3.05). The least motivating factor was being a researcher in the field of dentistry with a mean score of 1.73.

**Table 1:** Distribution (%) of the responses by Nigerian dental students (n = 179) to the presented motives to study dentistry, and the mean scores given to each motive

**Statements**	**Mean score**	**Frequency (%) of choices**					
		**0**	**1**	**2**	**3**	**4**	**5**
Helping people to improve their oral health	2.09	36.0	17.7	9.2	10.5	6.5	20.1
Playing a role in community health promotion	1.77	33.5	20.5	11.2	15.5	7.5	11.8
Being a researcher in the field of dentistry	1.73	32.1	22.2	6.8	15.4	11.1	12.4
Personal interest in dentistry	2.88	15.6	14.3	9.7	14.3	13.6	32.5
Interest in working with one’s hands	2.81	17.9	18.5	4.6	15.9	19.9	23.2
Existence of artistic theme in dentistry	2.26	27.4	16.0	10.2	17.2	10.2	19.1
High income from practicing dentistry	2.33	24.7	9.1	14.9	20.8	13.0	17.5
Ability to be self-employed	3.12	16.0	10.0	9.3	10.0	18.7	32.0
Social status of being a dentist	2.52	22.9	11.1	14.4	14.4	17.0	20.3
Parents’ recommendation	3.17	14.5	6.6	16.5	14.5	19.1	30.0
Friends’ or relatives’ recommendation	3.05	12.7	12.7	14.7	15.3	18.7	26.0
Failure to be admitted to other programmes	2.30	21.9	19.2	14.6	17.2	11.9	15.2

Table 2 lists the items that make up the four factors that resulted from the factor analysis. Based on the computed sum variables for these factors, two profession-related dimensions and two individual factors were identified. The two profession-related dimensions identified were Factor 1 which accounted for 41% of the variance and contained three items which focused on job security and social status. Factor 2 accounted for 31% of the variance and contained three items that focused on altruism and intellectual challenge. Factors 3 and 4 were related to individual factors namely ‘Existence of artistic theme in dentistry’ and ‘Parents’ recommendation’ respectively. Factors 3 and 4 contained single items that accounted for 21% and 17% of the explained variance in the data respectively. The reliability of the factors was high: 0.89 for Factor 1 and 0.83 for Factor 2.

**Table 2:** Factor loadings* on study motives amongst senior dental students in Nigeria (n = 179)

**Statements**	**Factors**			
	**1**	**2**	**3**	**4**
High income from practicing dentistry	*0.676	0.139	0.256	0.214
Ability to be self-employed	*0.746	0.342	0.205	0.013
Social status of being a dentist	*0.736	0.402	0.129	0.145
Helping people to improve their oral health	0.439	*0.633	0.172	0.008
Playing a role in community health promotion	0.336	*0.677	0.219	0.103
Being a researcher in the field of dentistry	0.373	*0.604	0.233	0.084
Existence of artistic theme in dentistry	0.269	0.332	*0.618	0.186
Parents’ recommendation	0.134	0.090	0.147	*0.659
Percentage of variance explained	0.41	0.31	0.21	0.17
Percentage of cumulative variance explained	0.41	0.72	0.93	1.11
Cronbach Alpha	0.89	0.83	-	**-**

1- Job security and social status; 2 – Altruism and intellectual challenges; 3 - Existence of artistic theme in dentistry; 4 - Parents’ recommendation.

By using the sum variables to identify the proportion of students who had each dimension as their highest ranked motive (Table 3), we found that the ‘characteristics of the profession’ was ranked first by 47.5% of the students followed by ‘altruism and intellectual challenge’ by 45.3% of the students. The mean scores for all four dimensions for both males and females, and for each age group were less than 3.0.

**Table 3:** Motive scale percentage distribution and mean for the final year dental students (n = 179) according to their four top-ranked motive to study dentistry and their background factors

**Motives**	**All (N = 179)**		**Sex**						**Age**							
			**Men (N = 106)**		**Women (N = 73)**		**P**		**< 27 yrs (n = 77)**		**27-29 yrs (n = 57)**		**> 29 years (n = 45)**		**P**	
	**%**	**Mean (SD)**	**%**	**Mean (SD)**	**%**	**Mean (SD)**	**%**	**Mean**	**%**	**Mean (SD)**	**%**	**Mean (SD)**	**%**	**Mean (SD)**	**%**	**Mean**
Characteristics of the profession	47.5	2.26 (1.72)	49.1	2.3 (1.78)	45.2	2.16 (1.63)	0.61	0.53	45.5	2.11 (1.78)	47.4	2.30 (1.65)	51.1	2.84 (1.71)	0.83	0.50
Altruism and intellectual challenges	45.3	2.31 (1.64)	50.0	2.43 (1.67)	38.4	2.12 (1.58)	0.12	0.21	36.4	2.09 (1.62)	47.4	2.32 (1.64)	57.8	2.67 (1.65)	0.07	0.17
Existence of artistic theme in dentistry	41.3	1.97 (1.90)	39.6	2.02 (2.00)	43.8	1.90 (1.77)	0.57	0.66	42.9	1.92 (1.87)	36.8	1.49 (1.70)	44.4	2.64 (2.05)	0.70	0.01
Parents’ recommendation	40.7	1.60 (1.74)	49.1	1.52 (1.66)	50.7	1.73 (1.84)	0.83	0.43	50.7	1.65 (1.75)	38.6	1.51 (1.72)	62.2	1.64 (1.76)	0.06	0.88

Further analysis showed that a lower number of females and respondents younger than 27 years chose ‘altruism’ and ‘intellectual challenges’ as a motives for the study of dentistry while a larger number of females had ‘Existence of artistic theme in dentistry’ as a motive for the study of dentistry. These observed sex and age differences were not statistically significant. There was no significant difference in the mean score on all the dimensions irrespective of sex. However, the mean score on the dimension ‘Existence of artistic theme in dentistry’ was significantly highest for respondents who were older than 29 years (p = 0.01).

### Career preferences among the students

As shown in Table 4, entering a postgraduate programme was the most popular career preference of the respondents (3.08): 67% of respondents showed a strong interest in entering for a postgraduate programme. Few respondents (35.1%) showed interest in practicing as a solo practitioner in a private clinic with the lowest mean of 1.80

**Table 4:** Distribution (%) of the responses by Nigerian senior dental students (n = 179) to the presented career preferences, and the mean scores given to each item

**Statements**	** Mean score**	**Frequency (%) of choices**					
		**0**	**1**	**2**	**3**	**4**	**5**
Solo practitioner in a private office	1.80	35.8	15.6	14.5	13.4	8.4	12.3
Shareholder in an incorporated practice	2.22	34.6	7.8	11.2	12.9	14.5	19.0
On salary in a government organisation	2.44	26.3	12.9	10.1	13.4	16.2	21.3
On salary/commission in a private organization	1.97	32.4	15.1	12.3	15.6	12.3	12.3
Work in the primary health care System	2.00	34.6	15.6	8.9	14.0	14.0	12.9
work or study in the field of oral public health	1.85	40.2	12.3	6.7	17.3	9.5	14.0
Be a researcher in dentistry	1.98	36.3	12.3	10.6	12.9	13.4	14.5
Entering a postgraduate programme	3.08	22.9	3.9	6.2	12.9	17.9	36.3

Based on the computed sum variables for these factors, one career preference dimension and one individual factor were identified: (1) Public oral health and research, and (2) Being a shareholder in an incorporated practice. Table 5 shows the percentage distribution of respondents with strong interest in each of the identified careers according to their background variables. No age or sex difference was observed in any of the career preferences.

**Table 5:** Percentage distribution of the final year dental students with strong interest in each career preference according to their background factors (n = 179)

**Career preference**			**Sex**						**Age**							
	**All**		**Men**		**Women**		**P**		**> 27 yrs**		**27-29 yrs**		**< 29 years**		**P**	
	**%**	**Mean (SD)**	**%**	**Mean (SD)**	**%**	**Mean (SD)**	**%**	**Mean (SD)**	**%**	**Mean (SD)**	**%**	**Mean (SD)**	**%**	**Mean (SD)**	**%**	**Mean (SD)**
Public oral health and research (To work as a dentist in the primary health care System; To work or study in the field of oral public health; To be a researcher in dentistry)	44.1	0.44 (0.50)	48.1	0.48 (0.50)	38.4	0.38 (0.49)	0.20	0.20	39.0	0.39 (0.49)	43.9	0.44 (0.50)	53.3	0.53 (0.50)	0.30	0.31
To be a shareholder in an incorporated practice	46.4	0.46 (0.50)	49.1	0.49 (0.50)	42.5	0.42 (0.50)	0.39	0.39	50.7	0.51 (0.50)	45.6	0.46 (0.50)	40.0	0.40 (0.50)	0.52	0.52

### Opinion about paediatric dental practice

Table 6 shows that majority of the respondents had positive opinions about paediatric dental practice. Over 70% of respondents felt the specialty was useful for the community (79.3%), valuable service (74.3%), efficient (73.8%), essential (72.7%) and scientific (71.5%). A little over half of the respondents (56.5%) however felt the practice of paediatric dentistry was expensive.

**Table 6:** Percentage distribution of final year students by their opinion about paediatric dental practice (n = 179)

	**1**	**2**	**3**	**4**	**5**	**6**	**7**	**9**
Useful for the community	1.7	1.1	1.7	3.4	2.8	7.8	68.7	12.9
Prestigious	5.6	3.4	4.5	7.8	5.6	12.3	45.3	15.6
Essential	4.5	-	2.8	3.9	1.7	11.2	59.8	16.2
Efficient	3.9	1.7	0.6	5.0	6.2	19.0	48.6	15.1
Scientific	4.5	1.1	1.1	7.3	10.1	14.5	46.9	14.5
Attractive	4.5	2.8	3.9	7.8	10.6	16.2	38.0	16.2
Valuable service	2.8	-	-	7.8	7.8	17.3	49.2	15.1
Simple	9.5	3.9	5.0	12.3	15.6	10.6	26.8	16.2
Not costly	14.0	2.8	6.7	17.3	14.5	7.8	21.2	15.6
Good use of dentist’s time	3.4	4.5	4.5	4.5	8.4	12.3	44.1	18.4

1 = negative opinion; 7 = positive opinion; 9 = no response.

Table 7 shows the percentage distribution of respondents based on the computed sum variables for these factors according to their background variables. Two opinion dimensions were identified: (1) Essence of paediatric dentistry, and (2) Perception of paediatric dental practice. There was no significant age or sex difference observed in any of the dimensions.

**Table 7:** Motive scale percentage distribution and mean distribution of the final year dental students with positive opinion about paediatric dental practice (n = 179)

**Opinion about paediatric dental practice**			**Sex**						**Age**							
	**All**		**Men**		**Women**		**P**		**> 27 yrs**		**27-29 yrs**		**< 29 years**		**P**	
	**%**	**Mean (SD)**	**%**	**Mean (SD)**	**%**	**Mean (SD)**	**%**	**Mean (SD)**	**%**	**Mean (SD)**	**%**	**Mean (SD)**	**%**	**Mean (SD)**	**%**	**Mean (SD)**
Essence of paediatric dentistry (Useful for the community, Prestigious, Essential, Efficient, Scientific, Attractive, Valuable)	49.7	0.50 (0.50)	50.0	0.49 (0.50)	49.3	0.51 (0.50)	0.93	0.83	50.7	0.5 (0.50)	42.1	0.4 (0.49)	57.8	0.6 (0.50)	0.28	0.14
Perception of paediatric dental practice (Simple, not costly)	48.0	0.37 (0.49)	47.2	0.40 (0.49)	49.3	0.34 (0.48)	0.78	0.47	48.1	0.38 (0.49)	43.9	0.32 (0.47)	53.3	0.44 (0.50)	0.64	0.41

### Motive for interest in paediatric dental practice

Table 8 shows the factors that serve as motive for interest for the 91 (50.8%) students who indicated interest in practicing paediatric dentistry. This included 61 (67.0%) of the male students and 30 (32.0%) of the female students. There was significantly more male students interested in the practice of paediatric dentistry (p = 0.03)

**Table 8:** Distribution (%) of the responses to the presented motives for interest in paediatric dentistry among Nigerian senior dental students with interest in paediatric dentistry (n = 91), and the mean scores given to each motive

**Statements**	**Mean score**	**Frequency (%) of choices**					
		**0**	**1**	**2**	**3**	**4**	**5**
Parent(s) practice paediatric dentistry	1.48	37.5	26.4	11.4	10.2	5.7	9.1
Friends’ or relatives’ practice paediatric dentistry	1.43	38.6	26.1	12.5	8.0	5.7	9.1
Personal interest in paediatric dentistry	2.44	25.0	12.5	11.3	17.1	12.5	21.6
General interest in working with children	2.67	14.8	17.1	20.4	9.1	11.4	27.3
Experience with working with children	2.47	20.5	14.8	12.5	19.3	15.9	17.1
High income from practicing paediatric dentistry	1.03	64.8	8.0	9.1	3.4	6.8	8.0
Ability to be self-employed	2.42	19.3	13.6	21.6	13.6	14.8	17.1
Social status of being a paedodontists	1.83	31.8	14.8	18.2	18.2	8.0	9.1
Helping children to improve their health	2.82	15.1	13.6	13.6	11.4	20.5	25.0
Becoming a researcher in the field of paediatric dentistry	2.43	17.1	19.3	15.9	15.9	14.8	17.1

The strongest motive was interest in helping children to improve their health (2.82) followed by general interest in children (2.67). About 65% of respondents did not agree that the practice of paediatric dentistry was financially lucrative.

Based on the computed sum variables for these factors, two motive dimensions were identified: (1) Personal interest, professional characteristic and interest of significant others in children which accounted for 65% of the explained variance in the data and contained six items and (2) Family influence which accounted for 25% of the explained variance in the data and contained two items. The reliability of the factors was high: 0.87 for ‘Personal interest, professional characteristic and interest of significant others in children’ and 0.82 for ‘family influence’. See Table 9.

**Table 9:** Factor loadings* on motives for interest in paediatric dentistry practice amongst senior dental students in Nigeria (n = 91)

**Statements**	**Factors**	
	**1**	**2**
Parent(s) practice paediatric dentistry	0.0919	*0.7638
Friends’ or relatives’ practice paediatric dentistry	0.0952	*0.7920
Personal interest in paediatric dentistry	*0.7929	0.1065
General interest in working with children	*0.8256	0.1122
Experience with working with children	*0.7475	0.2349
High income from practicing paediatric dentistry	0.5010	0.0561
Ability to be self-employed	0.5615	0.2746
Social status of being a paedodontists	*0.6394	0.1416
Helping children to improve their health	*0.7164	−0.1266
Becoming a researcher in the field of paediatric dentistry	*0.6145	0.1154
Percentage of variance explained	0.65	0.25
Percentage of cumulative variance explained	0.65	0.90
Cronbach’s Alpha	0.87	0.82

1- Personal interest, professional characteristic and interest of significant others in children; 2 – Family influence.

Table 10 shows the percentage distribution of respondents with strong interest in each of the identified motives for interest in paediatric dentistry according to their background variables. About 47% of the respondents identified ‘Personal interest, professional characteristic, and interest in significant others” as a motive for interest in paediatric dentistry while about 43% identified ‘family influences’ as a motive. There was no significant difference in the number of males and females, and in the number of respondents in each age group who identified any of the dimensions as a reason for interest in paediatric dentistry. Neither were there are differences in the mean scores of the sexes and each age group for each of the dimensions.

**Table 10:** Motive scale percentage distribution and mean for the final year dental students by motives for interest in paediatric dentistry practice according to their two top-ranked motive to study dentistry and their background factors background factors (n = 91)

**Motives**	**All (N = 91)**		**Sex**						**Age**							
			**Men (N = 61)**		**Women (N = 30)**		**P**		**< 27 yrs (n = 35)**		**27-29 yrs (n = 20)**		**> 29 years (n = 26)**		**P**	
	**%**	**Mean (SD)**	**%**	**Mean (SD)**	**%**	**Mean (SD)**	**%**	**Mean (SD)**	**%**	**Mean (SD)**	**%**	**Mean (SD)**	**%**	**Mean (SD)**	**%**	**Mean**
Personal interest, professional characteristics and interest of significant others in children	47.3	2.73 (1.25)	41.0	2.57 (1.32)	60.0	3.04 (1.05)	0.09	0.09	45.7	2.778 (1.29)	50.0	2.64 (1.14)	46.2	2.75 (1.37)	0.93	0.91
Family influence	42.9	1.42 (1.48)	42.6	1.41 (1.58)	43.3	1.45 (1.30)	0.95	0.90	42.9	1.46 (1.59)	40.0	1.37 (1.48)	46.2	1.44 (1.40)	0.90	0.97

## Discussion

The final year students were targeted for the study as we assumed that they have had adequate exposure to the practice of dentistry to be able to havean informed opinion about choices they make with respect to current and future dental practice.

The three top reasons for interest in the study of dentistry by dental students in Nigeria is motivation from parents, motivation from family and relatives, and the increased prospect to be self-employed. This is unlike what was observed with Iranian students who identified social status as a top ranking motive for choosing dentistry as a course [8]. Just like multiple authors had earlier highlighted [1-4,8], job security plays a big role in the choice of dentistry as a career. For a country like Nigeria where the social security system is poor, people seek to study professional courses where the prospect for self-employment is high.

The present study showed that a moderate number (45%) of students had altruism and intellectual challenges as their first motive dimension. This finding is contrary to reports about dental students in Iran [8] and Britain [3] for whom altruism and intellectual challenges were rated low motives for studying dentistry. The authors are not of the opinion that the curriculum of the dental schools in Nigeria lay emphasis on giving back to the society and thus, cannot readily explain the altruism reflected in the results of the present study. This finding may be attributed to the influence of the social environment and the culture in Nigeria: the high religiosity gives credence to being altruistic especially when it comes to discussions on health. Altruism is a recognized way of life and cultural practice in Nigeria and this determines the health condition, strength and activities of the people [24]. The observation may also be due to the desire of the respondents to choose socially acceptable responses, a limitation that has been observed in questionnaire surveys [25].

However, the training emphasizes the importance of postgraduate medical education which is synonymous with research work in the context of medical and dental education in Nigeria. This may be a reason for the observation in this study: postgraduate education was the most popular career preference and intellectual challenge was identified as a strong motivating factor for studying dentistry. This has certain implications for workforce planning for the country.

Similar to the observation reported by Khami et al. [8] and Hallissey et al. [4], there was no significant sex differences observed in motives for studying dentistry and career preferences. This observation is however at variance with those of other studies [10-12,14,15,23]. Prior studies have shown that sex and race shape student’s orientation [26]. The observation may be in line with reflections by Khami et al. [8], who noted that the differences in observation between studies and countries may be a reflection of the effect of cross-cultural and background differences in gender norms and practices between societies and countries. Though this study did observe subtle though insignificant sex differences in motives and career preferences, it may be important to further investigate the possible impact of culture and socialization on sex differences in study and career preferences in dentistry.

The opinion of respondents about paediatric dentistry was positive. There however were a number of students who had the impression that paediatric dental practice was not financially lucrative. Despite this impression, there were those who showed interest in the practice of paediatric dentistry as reflected in Table 9. The reason for this impression was not sought from the students during the data collection process. However, the authors observed that there are currently no private practices in Nigeria providing exclusive specialized paediatric oral health services unlike what obtains with orthodontics, restorative dentistry and oral and maxillofacial surgery practices. This may be a reason for the response and enough reason to dissuade dental students whose motive for studying dentistry was the potential to make high income. The potential to earn high income in Nigeria is better as an operator of a private practice than working in the public health service.

Over half of the respondents (50.8%) indicated interest in paediatric dentistry as a future career. Significantly more male students showed interest in the practice of paediatric dentistry. Newton et al. had earlier reported an increase in tendency of females to work as paediatric dentists [27]. Scarbecz and Ross [28] also reported a larger number of females entering the paediatric dentistry programme in the USA. Similarly, the number of females who specialized in paediatric dentistry in Nigeria exceeds the number of males. However, there are increasing number of more males employed for residency training in paediatric dentistry (Personal communication with the Nigeria Association of Paediatric Dentistry). With more male students showing interest in the practice of paediatric dentistry, it is likely that the number of males applying for residency training in paediatric dentistry in the future would continue to increase.

Motivating dimensions for interest in paediatric dentistry were ‘Personal interest, professional interest and interest of significant others in children’, and ‘family influence’. There were no sex or age related observed differences in the motives for interesteither based on the number of those who identified either of the dimensions as a motivating factor or the mean score on the dimensions. There is apparently a shift in the perception of paediatric dentistry as being aligned with culturally defined sex roles [26]. This may be a pointer to changing culture and socialization processes as a whole or an identified need by students to position themselves to get available jobs in the Dental Health industry. Currently, there are a number of dental schools that do not have paediatric dentists on their staff list implying that there is the potential for getting employment in the public health sector and the academia in the near future if they specialize in paediatric dentistry.

The study has its strengths and limitations. There was a high response rate thereby guaranteeing the target population being well represented in the study. However, a self-administered questionnaire was used as a survey instrument. This is a quick, practical, and economical way of data collection [29], though its use is associated with an increased tendency for positive and socially accepted responses [25]. Also, the section of the tool that measured motive for interest in the study of paediatric dentistry could have been tested for its content validity since this was the first time this tool was developed and used for such measure. The section is however an adaptation of a tool that had been used in a previous study to assess final year dental students’ motives for interest in dentistry [8]. This adaptation is applicable to assess interest in paediatric dentistry since the central theme of the measure was ‘interest’.

## Conclusion

Final year dental students in Nigeria were motivated to practice dentistry because of the characteristics of the profession, altruism and intellectual challenges, existence of artistic theme in dentistry and parent’s recommendation. The main motive for showing interest in the practice of paediatric dentistry were ‘personal interest, professional interest and interest of significant others in children’, and ‘family influence’. Significantly more males than females were interested in the practice of paediatric dentistry though the motives for interest in the practice of paediatric dentistry did not differ by sex or age: a possible reflection of changes in strong cultural themes in the motives for career choices in Nigeria.

## Competing interests

The authors declare that they have no competing interests.

## Authors’ contributions

MOF: conceived the idea of the paper. MOF, OOS, MRK, AOE, BOP, OOO, NF, TOL, all made substantial contributions to conception, design, acquisition, analysis and interpretation of data for this study; were involved in drafting and revising the manuscript for important intellectual content; and have given final approval of the version to be published. BP was involved with the data analysis and interpretation of data for this study; was involved in drafting and revising the manuscript for important intellectual content. All authors read and approved the final manuscript.

## Pre-publication history

The pre-publication history for this paper can be accessed here:

http://www.biomedcentral.com/1472-6920/14/130/prepub
